# 

**Published:** 1905-01

**Authors:** 


					﻿Sixtieth Year,
JANUARY, 1905.
BUFFALO
MEDICAL JOURNAL
VOL. LX.
Whole Number.
DCLXXXXVIII
New Series.
VOL. XLIV
No. 6. '
Established 1845 by AUSTIN FL1NT,*THT.D.
EDITOR :
WILLIAM WARREN POTTER,’ M. D.
ASSISTANT EDITORS:
WILLIAM C. KRAUSS, M. D.
NELSON W. WttBONv-Mr-D.-
ASSOC1ATE EDITORS:
JAMES WRIGHT PUTNAM, M. D.
JOHN PARMENTER, M. D.
HARVEY R. GAYLORD, M. D.
ERNEST WENDE, M. D.
JOHN A. MILLER, Ph. D.
MAUD J. FRYE, M. D.
For terms and other information relating to the Journal, see page vi.
				

